# Structure and Mechanisms of Assembly-Line Polyketide Synthases

**DOI:** 10.1146/annurev-biochem-080923-043654

**Published:** 2024-07-02

**Authors:** Alexander M. Soohoo, Dillon P. Cogan, Krystal L. Brodsky, Chaitan Khosla

**Affiliations:** 1 Department of Chemical Engineering, Stanford University, Stanford, California, USA;; 2 Department of Chemistry, Stanford University, Stanford, California, USA; 3 Sarafan ChEM-H, Stanford University, Stanford, California, USA; 4 Current affiliation: Department of Pharmacology and Pharmaceutical Sciences, University of Southern California, Los Angeles, California, USA

**Keywords:** polyketide, polyketide synthase, catalytic cycle, vectorial biosynthesis, multifunctional enzyme

## Abstract

Three decades of studies on the multifunctional 6-deoxyerythronolide B synthase have laid a foundation for understanding the chemistry and evolution of polyketide antibiotic biosynthesis by a large family of versatile enzymatic assembly lines. Recent progress in applying chemical and structural biology tools to this prototypical assembly-line polyketide synthase (PKS) and related systems has highlighted several features of their catalytic cycles and associated protein dynamics. There is compelling evidence that multiple mechanisms have evolved in this enzyme family to channel growing polyketide chains along uniquely defined sequences of 10–100 active sites, each of which is used only once in the overall catalytic cycle of an assembly-line PKS. Looking forward, one anticipates major advances in our understanding of the mechanisms by which the free energy of a repetitive Claisen-like reaction is harnessed to guide the growing polyketide chain along the assembly line in a manner that is kinetically robust yet evolutionarily adaptable.

## INTRODUCTION

Within the superfamily of evolutionarily related fatty acid synthases (FASs) and polyketide synthases (PKSs) (1–3), assembly-line PKSs comprise a class of unusually large multienzyme systems (1–10 MDa) that catalyze the biosynthesis of structurally and functionally diverse metabolites in eubacteria and eukaryotes. Many of these bioactive natural products are used as medicines in human and animal health, while others have illuminated fundamentally new biological mechanisms in prokaryotes or eukaryotes. As just one example, the polyketide rapamycin has not only been exploited as an immunomodulatory and anticancer drug but also inspired countless studies based on its ability to function as a molecular glue that noncovalently brings together two otherwise noninteracting proteins in mammalian cells (4).

**Figure 1:**
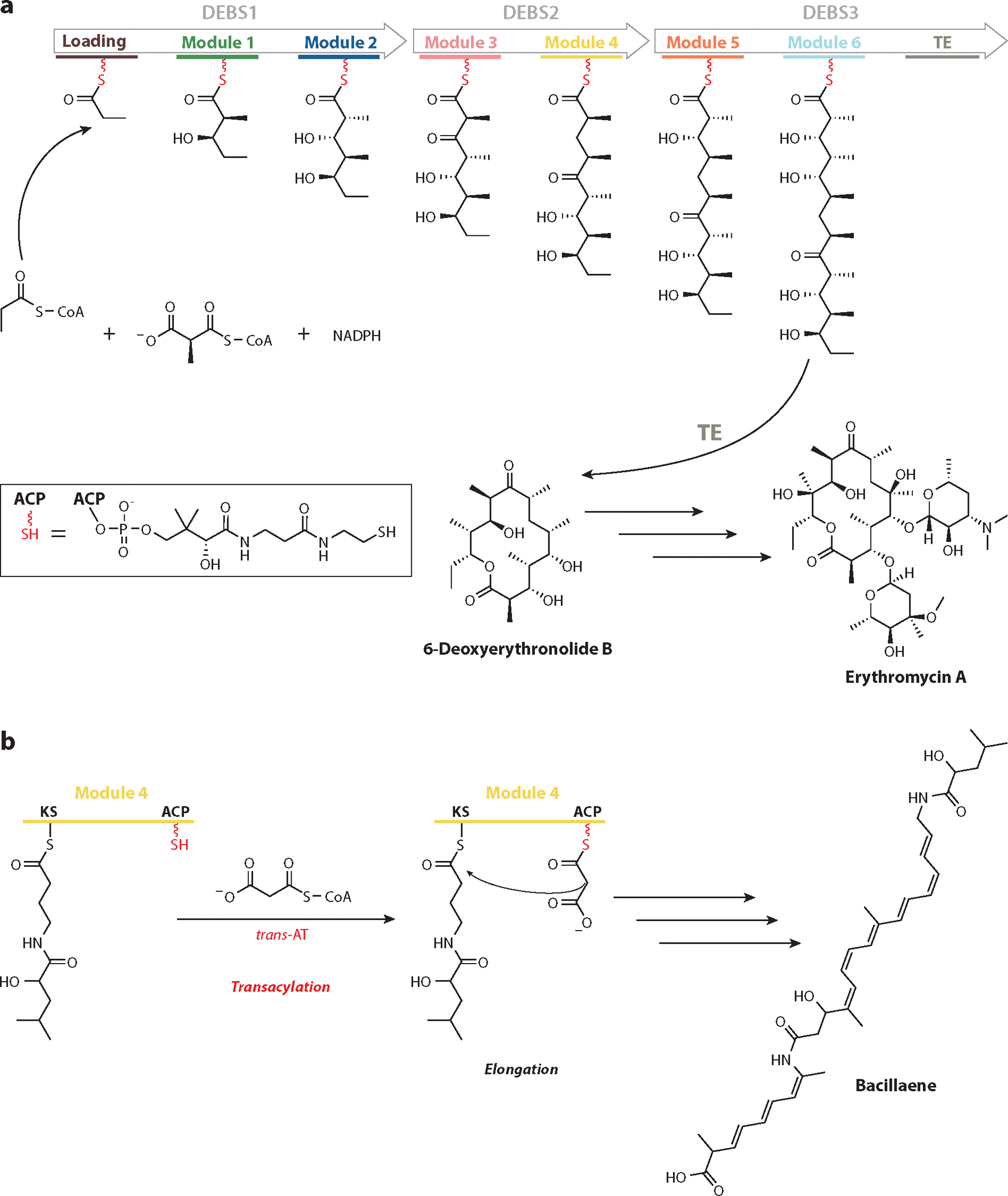
PKS biosynthesis. (*a*) DEBS. Synthesis of 6-deoxyerythronolide B, the macrocyclic precursor of the antibiotic erythromycin, requires one equivalent of propionyl-CoA, six equivalents of methylmalonyl-CoA, and six equivalents of NADPH. The DNA sequences of three unusually large genes (~10 kb each, encoding proteins designated DEBS1, DEBS2, and DEBS3) predicted the existence of a remarkably elegant architecture based on its similarity to the domains of a typical vertebrate fatty acid synthase (5, 6). Specifically, the inferred assembly-line PKS consists of a loading module that utilizes propionyl-CoA, followed by six elongation modules that utilize one methylmalonyl-CoA equivalent each and a terminal TE domain that releases the fully elaborated polyketide chain via concomitant macrocyclization. Each elongation module harbors a KS, AT, and ACP domain, along with one or more optional domains homologous to NADPH-dependent KR, DH, and NADPH-dependent ER domains from fatty acid synthases. For example, Modules 1, 2, 5, and 6 harbor only a KR domain, whereas Module 4 has a full complement of KR, DH, and ER domains. The domain composition of each module enables sequential elaboration of the growing polyketide chain, with each module catalyzing one chain-elongation cycle along with the requisite postelongation chemical modifications that give rise to the functionality and stereochemistry observed at the corresponding backbone carbon atoms of the polyketide natural product. (*b*) The bacillaene synthase is a *trans*-AT PKS in which a discrete AT protein (*trans*-AT) partners with AT-less modules, such as Module 4, to catalyze transacylation of a malonyl extender unit onto the ACP. The ACP-bound malonyl extender is then converted into an enolate nucleophile that attacks the acyl thioester bound at the KS active site, constituting one round of chain elongation during bacillaene biosynthesis. Abbreviations: ACP, acyl carrier protein; AT, acyltransferase; DEBS, 6-deoxyerythronolide B synthase; DH, dehydratase; ER, enoylreductase; KR, ketoreductase; KS, ketosynthase; PKS, polyketide synthase; TE, thioesterase.

Since the first reports of the extraordinary multimodular architecture of the 6-deoxyerythronolide B synthase (DEBS) (Figure 1a) (5, 6), there has been a steady growth in the number of sequenced assembly-line PKSs that synthesize diverse polyketides such as macrolides, polyene and polyether antibiotics, aromatic compounds, and metabolites of mixed polyketide and nonribosomal peptide origin (7, 8). As of our last census of public databases (9), more than 400 assembly-line PKSs involved in the biosynthesis of structurally characterized natural products have been cloned and sequenced. During the same period, however, whole-genome sequencing has led to explosive growth in the identification of orphan assembly-line PKSs whose metabolic products are unknown (9–11). Indeed, sequences of more than 8,000 nonredundant orphan assembly-line PKSs are publicly available (https://orphanpkscatalog2022.stanford.edu/catalog); many of them come from nontraditional biological niches (12), while others reveal novel enzyme chemistry (8). Until our understanding of assembly-line PKS structure and mechanism achieves considerably greater predictive power, this gap between known and unknown polyketide natural products is likely to continue to grow.

Here, we review our current understanding of the mechanistic logic of polyketide biosynthesis on enzymatic assembly lines with a special focus on the defining features of this unusual metabolic paradigm. Above all, our assembly-line metaphor for these multienzyme systems directly challenges perceptions of this PKS family as clusters of enzymes operating in proximity to afford restricted diffusion of intermediates between successive active sites. As elaborated below, while restricted diffusion is necessary due to the covalently bound nature of biosynthetic intermediates, evolution has introduced other mechanisms to coordinate the turnover of spatially separated active sites along the assembly line. As such, this PKS family is more appropriately analogized to a highly programmed automotive assembly line than to the neatly lined shelves of a grocery store that can be conveniently sampled by individual shoppers without significant requirements for coordination between customers. This focus of our review precludes an ability to dive deeply into the evolution (10), architecture (13, 14), or engineering (15) of assembly-line PKSs, for which readers are directed elsewhere.

## HALLMARKS OF ASSEMBLY-LINE POLYKETIDE SYNTHASES

### Modularity

As illustrated by DEBS (Figure 1a), arguably the most salient feature of assembly-line PKSs is their modular architecture, which implies the existence of inbuilt mechanisms for ensuring that the growing polyketide chain is channeled along a uniquely defined sequence of modules, each used only once in the overall catalytic cycle of the PKS. (This directional aspect of polyketide synthesis via an assembly-line PKS is hereafter referred to as vectorial biosynthesis.) Notably, although the module order in DEBS is collinear with the sequence of biosynthetic events involved in 6-deoxyerythronolide B (6-dEB) biosynthesis, collinearity is neither an invariant feature of this enzyme family nor is it essential for assembly-line PKS function. For example, although the loading didomain, Module 1, and Module 2 of DEBS are encoded within the same polypeptide in their order of usage, they can be expressed as three stand-alone proteins without qualitatively or quantitatively altering the biosynthetic outcome of DEBS (16). In rare instances, individual modules can be either utilized more than once (a phenomenon known as stuttering) or omitted from the overall catalytic cycle (a phenomenon known as skipping) (17).

**Figure 2:**
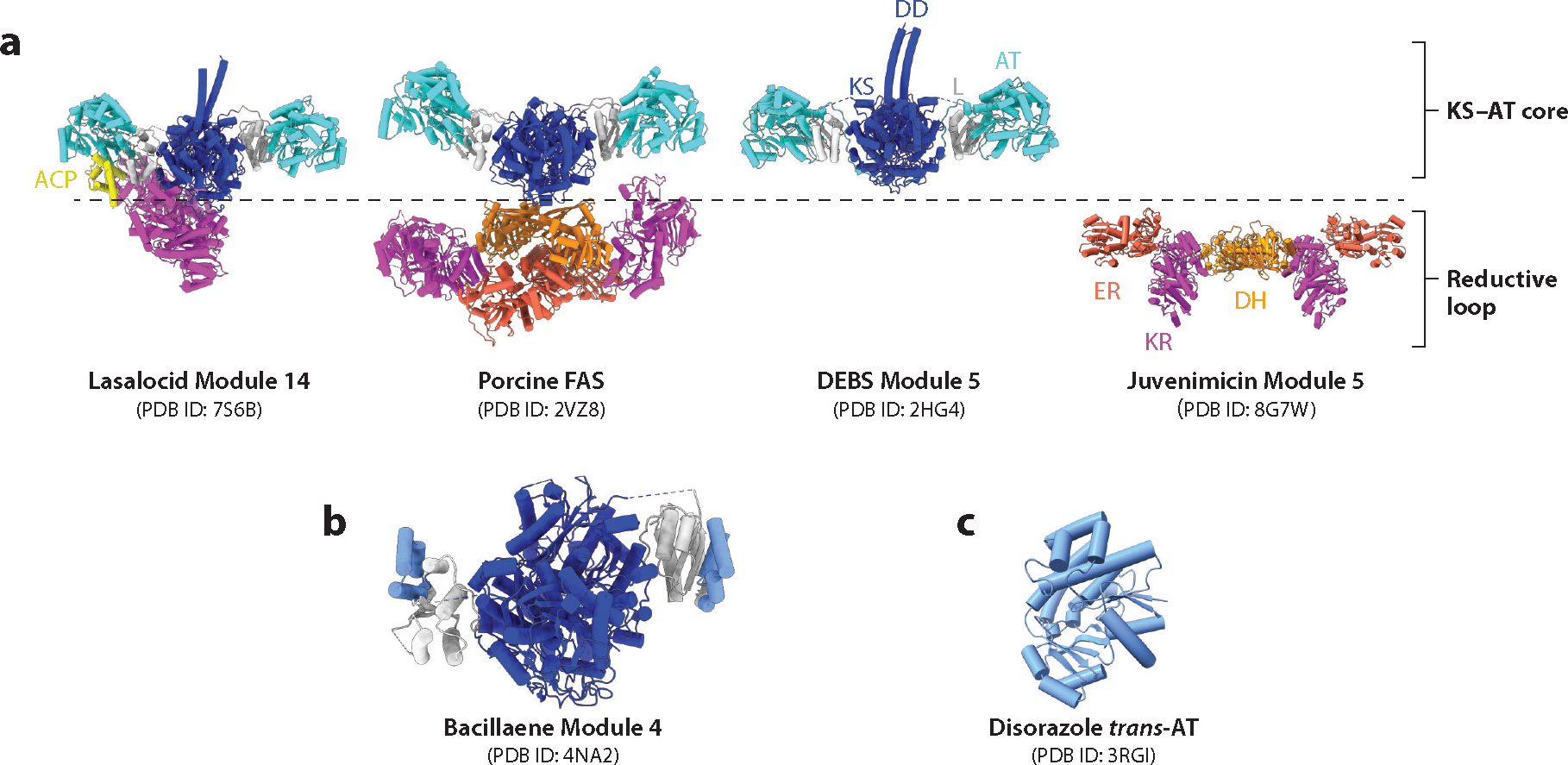
PKS module architecture. (*a*) The evolutionarily related PKSs and FASs, represented by the lasalocid PKS (56) and the porcine FAS (84), share a similar domain architecture. Individual domain types are displayed in the same color across all proteins in this panel. While the KS–AT core (27) is well conserved between the PKS and FAS systems, the architecture of the reductive loop is variable. FASs contain a reductive loop consisting of a DH, KR, and ER domain, whereas assembly-line PKS modules can have reductive loops consisting of only a subset of (e.g., lasalocid Module 14) or all three (e.g., juvenimicin Module 5) domain types (55). The DD and L domains are also common features of PKS modules. (*b*) Structure of the KS core of a *trans*-AT module from the bacillaene synthase (53). The LINKS motifs appended to the KS–AT linker are shown in sky blue. (*c*) Structure of the *trans*-AT involved in disorazole biosynthesis (73). Abbreviations: ACP, acyl carrier protein; AT, acyltransferase; DD, docking domain; DEBS, 6-deoxyerythronolide B synthase; DH, dehydratase; ER, enoylreductase; FAS, fatty acid synthase; KR, ketoreductase; KS, ketosynthase; L, KS–AT linker domain; LINKS, laterally interacting ketosynthase sequence; PDB ID, Protein Data Bank identifier; PKS, polyketide synthase.

The architectural features of a typical PKS module are illustrated in Figure 2a, where they are also compared to those of a mammalian FAS. While the core of a PKS module [composed of its ketosynthase (KS) and acyltransferase (AT) domains] is very similar to its FAS counterpart, the architectures of the reductive loops [consisting of the ketoreductase (KR), dehydratase (DH), and enoylreductase (ER) domains] of the two enzyme families are more divergent.

Whereas each of the six module cores of DEBS bears a strong resemblance to the core of a mammalian FAS, many assembly-line PKSs harbor AT-less modules. These systems are referred to as *trans*-AT PKSs, as illustrated by Module 4 of the bacillaene synthase (Figure 1b), because multiple AT-less modules share a stand-alone AT protein that is typically coexpressed with the assembly-line PKS. The architectures of Module 4 of the bacillaene synthase as well as the stand-alone AT from the disorazole PKS are shown in Figure 2b and c, respectively. They reveal close similarities between the KS and KS–AT linker domains of *trans*-AT PKS modules and their *cis*-AT counterparts (e.g., DEBS modules), while also highlighting the requirement for a potential docking site in the former type of module for a stand-alone AT.

As more and more assembly-line PKSs have been characterized, much has been learned about the structural and functional diversity of PKS modules. These insights have reinforced the notion of a module as a fundamental catalytic unit in assembly-line polyketide biosynthesis, prompting the assumption that assembly-line PKS evolution was heavily driven by intermodular recombination events. While this hypothesis, if correct, has immense engineering implications, evidence suggests that evolutionary diversification may have also leveraged crossover sites located within module boundaries (18). Presumably, these evolutionarily successful recombination events were enabled by the architectural flexibility of the reductive loop of a module, as illustrated in Figure 2a.

### Catalytic Cycle of a Polyketide Synthase Module

**Figure 3:**
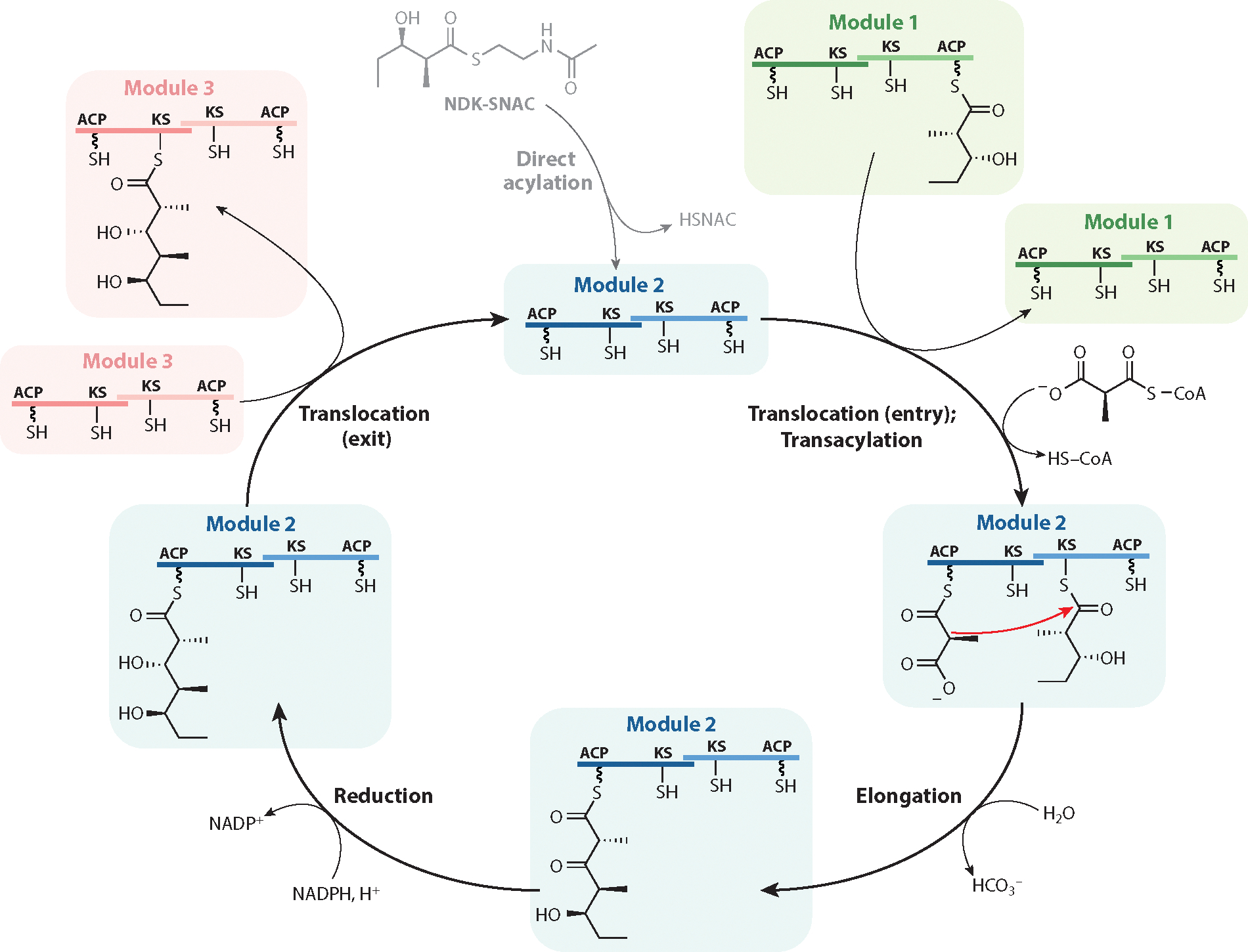
The catalytic cycle of DEBS Module 2. Starting from the entry translocation step, the upstream Module 1 passes its ACP-bound product to the KS domain of Module 2. Because Modules 1 and 2 are encoded within the same protein, this reaction is known to occur in an intrapolypeptide manner (i.e., involving ACP and KS domains from the same subunit of the homodimeric PKS) (24). In the absence of a suitable donor ACP, the entry translocation reaction can be enabled by an *N*-acetyl-cysteamine substrate mimic, such as NDK-SNAC. A transacylation reaction catalyzed by the AT domain of Module 2 installs a (2*S*)-methylmalonyl-CoA-derived extender unit onto the ACP domain of this module, although the relative timing of transacylation and entry translocation is unclear. Chain elongation occurs through an intermolecular decarboxylative Claisen condensation reaction, elongating the polyketide chain and stationing it on the ACP of the other subunit (i.e., elongation occurs in an intersubunit manner). The triketide product is then reduced by the KR domain in this module, whereafter it is passed to the KS domain of Module 3 via the exit translocation reaction. Note that the growing polyketide chain crosses the dimer interface over the course of the catalytic cycle (the two subunits of Module 2 are shown in distinct shades of blue). Abbreviations: ACP, acyl carrier protein; AT, acyltransferase; CoA, coenzyme A; DEBS, 6-deoxyerythronolide B synthase; KR, ketoreductase; KS, ketosynthase; NDK-SNAC, *N*-acetyl-cysteamine thioester of the natural diketide intermediate; PKS, polyketide synthase.

The catalytic cycle of a typical module includes three invariant types of reactions, two of which are shared by all FASs and PKSs, while the third has aspects that are unique to assembly-line PKSs. To illustrate these properties, the catalytic cycle of Module 2 of DEBS (whose biosynthetic role is defined in Figure 1a) is shown in Figure 3.

All PKSs and FASs catalyze two universal reactions. An acyltransferase(AT) catalyzes a thiol-to-thioester exchange that moves a unique α-carboxyacyl building block from an acyl-CoA substrate onto the acyl carrier protein (ACP) domain. As discussed above, this AT-catalyzed reaction (hereafter referred to as transacylation) can be performed either in *cis* or in *trans*. The other universal reaction (hereafter dubbed elongation) is the signature decarboxylative Claisen-like condensation that leads to polyketide chain growth via C–C bond formation. Importantly, this is the principal exergonic step within the catalytic cycle of a module.

While transacylation and elongation operate in a conserved fashion across all FASs and PKSs, the uniqueness of assembly-line PKSs stems from another thiol-to-thioester exchange reaction that underpins vectorial polyketide biosynthesis. This reaction (hereafter referred to as translocation) occurs twice in the catalytic cycle of a typical module and involves the engagement of distinct pairs of KS and ACP domains. As shown in Figure 3, the KS domain of DEBS Module 2 receives its diketide substrate from the ACP domain of Module 1 (labeled as the entry translocation step), while the triketide product of Module 2 is translocated from its ACP domain to the KS domain of Module 3 (labeled as the exit translocation step).In all other PKSs and FASs, elongation and translocation involve back-and-forth movement of the growing polyketide chain between the same KS–ACP pair of a single iteratively used module of catalysts. Looking at it differently, the KS active sites of FASs and iterative PKSs hold onto the free end of their growing fatty acid or polyketide chain through the entire duration of its biosynthesis. Biochemical evidence has confirmed that, while the tethered end of the growing chain toggles between KS and ACP thiols, an inbuilt mechanism in these iterative systems allows the ACP to back-translocate the growing chain to the same KS after each elongation cycle (19). In contrast, the two distinct translocation steps in the catalytic cycle of assembly-line PKS modules (Figure 3) necessitate extraction of each newly elongated polyketide intermediate from the corresponding KS active site followed by subsequent reinsertion into the KS active site of the next module. This behavior of assembly-line PKS modules is analogous to the classic drinking bird toy (where the bird’s beak represents the ACP and the glass of water represents the KS active site) and must have been an ancient evolutionary feat as assembly-line PKSs were evolving from their FAS-like ancestors. Its mechanistic underpinnings remain a virtual mystery as of now.

In addition to transacylation, elongation, and translocation, many PKS modules catalyze one or more optional modifications on the β-ketoacyl product of the elongation reaction. For example, DEBS Module 2 catalyzes reduction of the β-ketoacyl–ACP product of the elongation step prior to its translocation to Module 3, whereas Module 4 harbors a DH and an ER domain in addition to a KR; together the three enzyme domains reduce the β-ketoacyl product of elongation into a fully saturated moiety (Figure 1a). By now, a spectacular diversity of enzymatic chemistry has been uncovered in the reductive loops of naturally occurring assembly-line PKSs, including cofactor-dependent reactions such as those involving pyridoxal 5-phosphate, flavin nucleotides, and *S*-adenosylmethionine (20).

### The Dimer

Another invariant property of PKS modules is their homodimeric nature (21–23), which is enforced by the propensity of all KSs to dimerize via an extensive intersubunit interface (although many individual PKS modules also possess secondary, less-conserved dimerization sites). Recognition of the dimeric architecture of assembly-line PKSs prompts at least three basic mechanistic questions. First, does the catalytic cycle of a PKS module operate within the confines of individual subunits, or is intersubunit collaboration possible? Second, while sequential module action is undeniable, is there a unique sequence of active site usage within a module as it proceeds through the catalytic cycle shown in Figure 3, or are alternative reaction coordinates involving equivalent but distinct active sites feasible? Third, do pairs of active sites of a homodimeric module show positive, negative, or no cooperativity? Answers to these fundamental questions are now at hand, at least in the context of DEBS.

To address the first two questions, experiments involving half-of-site activity analysis were performed with DEBS. They revealed that, whereas polyketide chain elongation required the collaboration of KS and ACP domains from opposite subunits of the dimer (24), AT-catalyzed transacylation of a methylmalonyl building block onto the ACP could occur in a dimer-agnostic manner (25).Interestingly, in situations where successive modules are covalently fused, half-of-site activity analysis revealed that intermodular chain translocation occurred within the same polypeptidechain(24).Forexample,translocationofthediketidefromtheACPdomainofModule1tothe KS domain of Module 2 occurs within the same subunit of DEBS1 (Figure 1a). Thus, the growing polyketide chain appears to zigzag across the intersubunit interface of DEBS; this feature is also illustrated in Figure 3.

While in vitro reconstitution of DEBS was necessary and sufficient to answer the first two questions posed above, the question of cooperativity remained elusive until the advent of single-particle cryo–electron microscopy (cryo-EM) analysis of PKSs and is therefore discussed below after a recap of our present understanding of DEBS structure, as enabled by cryo-EM analysis.

### The Phosphopantetheine Arm

The final hallmark of assembly-line PKSs meriting attention is the presence of a 4′-phosphopantetheine (Ppant) arm posttranslationally attached to the ACP domain of each module within the multienzyme system (Figure 1a). The Ppant arm is appended to a conserved serine, converting the inactive *apo* protein to its *holo* form using coenzyme A (CoASH) as a substrate. This reaction is catalyzed by the phosphopantetheinyl transferase (PPTase) enzyme family (26). The Ppant arm facilitates movement of the polyketide chain between different domains during the catalytic cycle. When fully stretched, the Ppant arm is ~18 Å, allowing it to reach into every catalytically active site of the module. However, because the arm length is considerably shorter than the distance between the active sites of a module, large-scale domain motion is essential for assembly-line polyketide biosynthesis (27). For example, the KS and AT active sites of the core module are ~80 Å apart (Figure 2a), implying dynamic flexibility of the ACP and/or KS–AT core between successive transacylation and elongation reactions of a module (Figure 3).

Enzymatic addition of polyketide substrates by the promiscuous Sfp PPTase from *Bacillus subtilis* has proven invaluable in studying PKSs (28). The structure, activity profile, and applications of PPTases are well chronicled (29).

## METHODS TO STUDY POLYKETIDE SYNTHASES

The field of assembly-line PKS discovery, analysis, and engineering has benefitted immensely from a steady influx of new tools from microbial genetics and bioinformatics as well as chemical and structural biology. While a thorough discussion of these technological advances is beyond the scope of this review, a few methods warrant brief discussion due to their special relevance to the insights discussed here.

### Expression Hosts for Polyketide Synthases

Although many important insights into polyketide biosynthesis have emerged through manipulation and analysis of assembly-line PKSs in native hosts, the low levels of protein expression and the challenges associated with genetic manipulation of poorly studied microorganisms present serious difficulties for biochemical analysis of PKSs from native hosts (30). The development of a plasmid-based heterologous expression system for large assembly lines in *Streptomyces coelicolor* (31) has enhanced access to multienzyme systems such as DEBS. More recently, heterologous expression systems in *Escherichia coli* have further simplified access to these proteins. Although DEBS can be expressed in *E. coli*, it is inactive because *E.coli* lacks a compatible PPTase (21).Characterization of the promiscuous Sfp PPTase proved to be a general solution for posttranslational modification of ACP domains of assembly-line PKSs expressed in *E. coli* (28).

An ideal *E. coli* heterologous expression strain would not only express a compatible PPTase but also supply atypical substrates such as propionyl-CoA and (2*S*)-methylmalonyl-CoA. To address these problems, *sfp* and *prpE*, encoding a propionyl-CoA synthetase, were integrated into the chromosome of *E. coli* under control of an IPTG-inducible promoter, generating the *E. coli* BAP1 strain (32). PrpE provides a source of intracellular propionyl-CoA in the presence of exogenous propionic acid. When BAP1 is transformed with plasmids encoding DEBS1, DEBS2, DEBS3, and a propionyl-CoA carboxylase for (2*S*)-methylmalonyl-CoA production, the resulting strain not only serves as a robust source of functional proteins but also is able to synthesize 6-dEB in the presence of exogenous propionic acid.

### In Vitro Reconstitution of Assembly-Line Polyketide Synthases

Early in vitro studies of reconstituted DEBS components relied on isolation and purification of these large proteins from *Saccharopolyspora erythraea* (33) (the native host for erythromycin production) and *S. coelicolor* (22). Among many noteworthy insights thus derived, these studies highlighted the importance of buffer composition for preserving the homodimeric integrity and especially the polyketide synthetic capacity of DEBS. In particular, the strong correlation between turnover rate and phosphate concentration in the assay buffer highlighted a potential role for hydrophobic interactions in maintaining quaternary structure integrity (34). More recently, citrate buffers have been shown to be even more effective at preserving turnover rate, thermal stability, and structural integrity (35). The mechanism by which these polyvalent anions enhance catalytic activity is not understood, although this effect has also been observed with assembly-line PKSs other than DEBS.

The advent of *E. coli*–based expression systems for assembly-line PKSs also broadened the scope of in vitro reconstitution to include individual modules and multimodular systems from other antibiotic biosynthetic pathways including, for example, rifamycin (36), epothilone (37), and pikromycin (38). More recently, in vitro reconstitution has also been shown to be effective at deorphanizing assembly-line PKSs whose natural products are unknown, such as the complete 3-MDa nocardiosis-associated polyketide synthase (39, 40).

### *N*-Acetyl-Cysteamine Thioesters as Substrates

Even before the discovery of assembly-line PKSs, antibiotic pathways subsequently shown to harbor these multimodular systems were found to accept synthetic mimics of advanced biosynthetic intermediates, so long as they were supplied as cell-permeable *N*-acetyl-cysteamine (NAC) thioesters to growing cultures of native host organisms (41, 42). Based on structural mimicry of a Ppant arm by NAC, it was presumed that these acyl chain substrates were incorporated into the polyketide natural product due to a thiol-to-thioester exchange reaction onto the active site Cys of an enzyme resembling the KS component of a typical FAS, but the identity of the actual acceptor enzyme remained unknown until the assembly-line biosynthetic paradigm was revealed.

Today, knowledge of the assembly-line PKS sequence along with the structure of its polyketide product allows prediction of the identity of each ACP-bound intermediate with high confidence. If a biosynthetic intermediate can be chemically synthesized as a free acid, its corresponding NAC thioester is generally capable of transacylating its cognate acceptor KS domain, although the apparent *K*_M_ for this nonphysiological translocation is orders of magnitude higher than that of the corresponding ACP thioester. For example, the NAC thioester of the natural diketide (NDK) intermediate in 6-dEB biosynthesis (hereafter referred to as NDK-SNAC) (Figure 3) is selectively converted into 6-dEB by any host cell expressing catalytically competent DEBS Modules 2–6 (including the TE domain), based on its ability to be recognized by the KS domain of DEBS Module 2 (43). Whereas the *K*_M_ of this KS domain for NDK-SNAC is ~1 mM, the same enzyme has a *K*_M_ of ~10 μM for the corresponding ACP-bound substrate (44, 45). Analogous synthetic thioesters (46,47) are also selectively accepted as competent substrates by KS domains of a number of other assembly-line PKSs. Importantly, due to the relative ease of synthesis of NAC thioesters and their cell-permeable character, such an approach has immense preparative power for the chemo-biosynthesis of unnatural polyketides.

### Chemo-Enzymatic Preparation of Acyl Carrier Protein Domains with Unnatural Prosthetic Arms

The discovery of the broad specificity of the Sfp PPTase for both its protein and cofactor substrates has prompted extensive use of this enzyme for installing unnatural Ppant analogs onto ACP domains of assembly-line PKSs. The basic strategy involves chemo-enzymatic synthesis of the CoA analog of the desired posttranslational modification, followed by Sfp-catalyzed installation of the unnatural prosthetic arm onto a target ACP. Two types of modifications have been particularly useful. The first involves installing a chemically modified and/or isotopically labeled acyl chain onto an ACP whose fate can then be followed through one or more downstream steps on the PKS assembly line (37). Alternatively, the Ppant arm can be replaced with a bio-orthogonal analog such as an electrophilic moiety that can cross-link to a nucleophilic residue in the active site onto which the ACP docks (48). The latter approach has been crucially important for analyzing selective KS–ACP interactions during individual steps of FAS and PKS catalytic cycles (49, 50).

### Dissociating Polyketide Synthase Modules into Functional Catalytic Components

**Figure 4:**
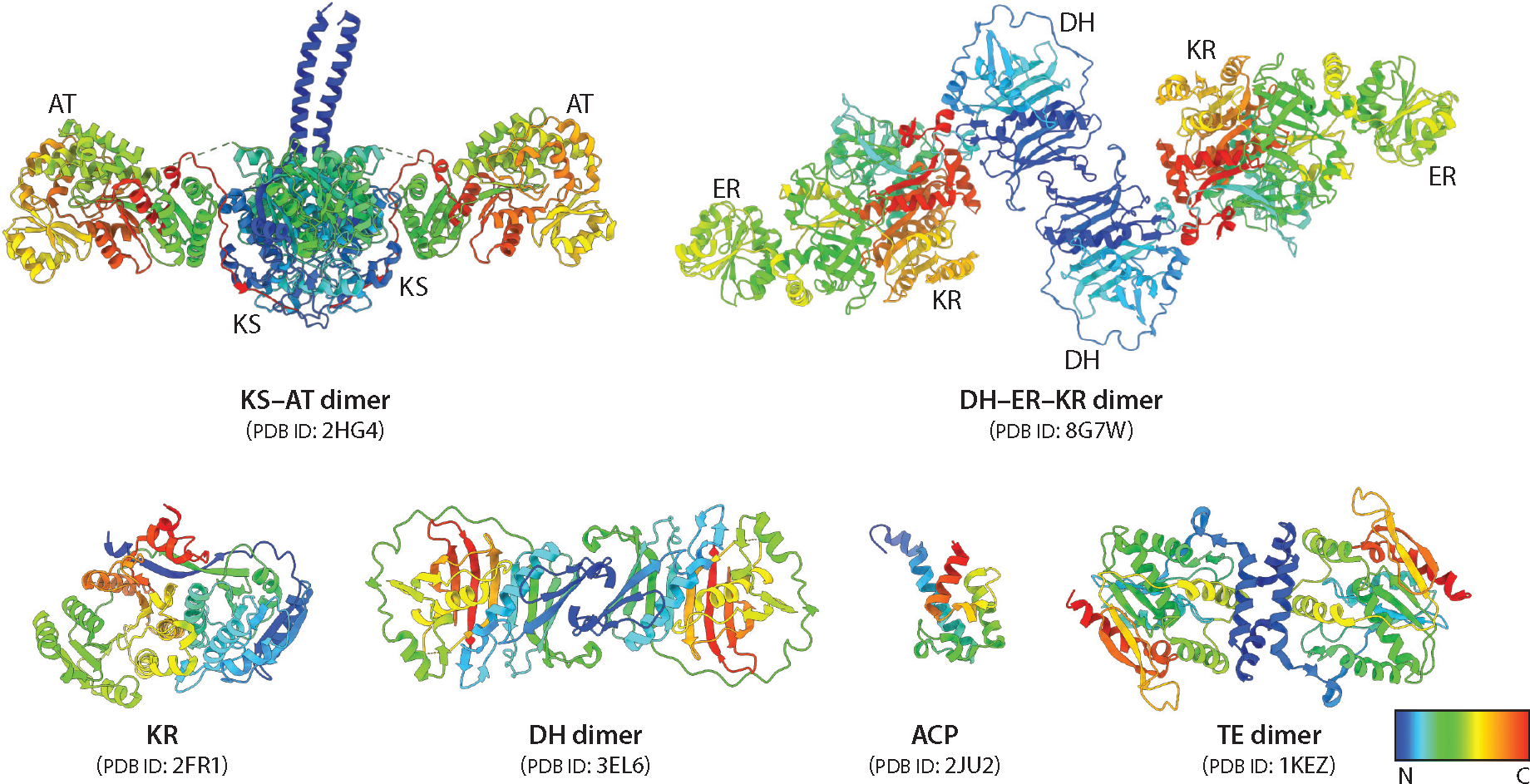
Structures of individual components of a PKS. Individual components of a PKS are shown. The KS–AT dimer (27), DH–ER–KR dimer (55), KR (54), DH dimer (74), ACP (76), and TE dimer (78) have been structurally characterized via X-ray crystallography (KS–AT, DH–ER–KR, KR, DH, and TE) or solution NMR (ACP). Structures are colored with a rainbow color scheme (N to C gradient, beginning with a dark blue). Abbreviations: ACP, acyl carrier protein; AT, acyltransferase; DH, dehydratase; ER, enoylreductase; KR, ketoreductase; KS, ketosynthase; NMR, nuclear magnetic resonance; PDB ID, Protein Data Bank identifier; PKS, polyketide synthase; TE, thioesterase.

Early attempts to express individual domains of assembly-line PKSs often yielded insoluble proteins, presumably due to an inability to accurately predict domain boundaries. To address this problem, limited trypsinolysis of DEBS Module 3 identified a highly conserved YRVDW sequence at the C-terminal end of the AT domain and another conserved RLAGL sequence near the N terminus of the ACP domain (51). Soon thereafter, structure-based analysis led to the identification of an acidic, Pro-rich sequence immediately downstream of KS domains (EEAPERE in DEBS Module 3) at which the KS and AT domains could be decoupled (52). The boundaries used to express *cis*-AT PKSs have also been used to functionally express the KS from *trans*-AT modules (53). To date not only have these domain boundaries found broad utility for heterologous expression of functional KS–AT didomains and KS, AT, KR (54), DH–ER–KR (55), and ACP (44) domains as stand-alone proteins, but also the resulting proteins interact with each other to promote the predicted elongation and postelongation reactions (Figure 4). In turn, structural and functional studies of these individual proteins and combinations thereof have contributed immensely to our present-day understanding of assembly-line PKS architectures and mechanisms. For example, as discussed below, decoding the stereodiversity of reductive loop enzymes required general approaches to physically separate the KS–AT–ACP core of modules from their KR, DH, and/or ER domains.

### Antibody Fragments as Conformational Traps of Short-Lived States of the Catalytic Cycle

The use of conformationally selective antibody fragments (F_ab_s) has enabled structural and mechanistic analyses of assembly-line PKS modules (35, 56). F_ab_s can trap a module at different points in the catalytic cycle by selecting and locking a transient state that might otherwise have too short of a lifetime to be amenable to structural analysis. One F_ab_ in particular, 1B2, acts like a molecular clamp, wrapping around the N-terminal coiled-coil docking domain of DEBS Module 3 (*K*_D_ ~ 100 nM), wherein each of two copies of 1B2 recognizes both subunits of the homodimeric module as well as one symmetry-related copy of itself (57). Although 1B2 is inhibitory to polyketide translocation mediated by intermodular docking interactions (presumably by competing for the same interface), it showed no inhibitory effect on the turnover of an isolated module limited by the rate of polyketide elongation. This feature, combined with its ability to uniformly promote the formation of module homodimers, has made 1B2 an invaluable, noninvasive probe for the structure–function analysis of intact PKS modules. The structural simplicity of the epitope recognized by 1B2 (the N-terminal docking domain of DEBS Module 3) also makes this approach readily applicable to the analysis of a variety of systems, as docking domains can readily be N-terminally fused with any PKS module of interest. Other F_ab_s have also found use in structural and mechanistic analyses of DEBS modules. For example, AA5 has been used as a crystallography chaperone for the KS–AT core of DEBS Module 2 (58), 1D10 and 2G10 have served as conformational probes of DEBS Module 1 (59), and 3A6 (60) was found to recognize the TE domain of DEBS without affecting module activity. This property of 3A6 makes it potentially useful as an inert tracer of TE movement without impeding overall catalysis.

## STRUCTURAL ANALYSIS OF ASSEMBLY-LINE POLYKETIDE SYNTHASES

As of the time of writing our last *Annual Reviews* article on this subject (61), prototypical high-resolution (below 3 Å) structures of individual domains and multidomain fragments from DEBS had just been solved, predominantly via X-ray crystallography. Representative structures of these PKS components are illustrated in Figure 4 and described below. Although X-ray crystallography continues to be invaluable for elucidating atomic-level structures of assembly-line PKS components that either are inherently rigid or can be coaxed into rigid conformations, other complementary tools are proving to be more useful for visualizing the structures of larger, more flexible portions of these systems at moderate resolution or, using advanced technologies, even near-atomic resolution. Specifically, size exclusion chromatography–small angle X-ray scattering (SEC-SAXS) and single-particle cryo-EM have been used to interrogate assembly-line PKSs with considerable impact, as summarized below.

### Docking Domains

Docking domains were discovered based on sequence analysis of DEBS modules (62). For example, the C terminus of Module 2 docks with the N terminus of Module 3 but not the N terminus of Module 5 (63). Because of the kinetic penalty due to mismatched or absent docking domains, it soon became clear that compatible docking domains were necessary to enable efficient vectorial polyketide biosynthesis across intermodular junctions (64). Mutagenesis and solution nuclear magnetic resonance (NMR) studies established that N-terminal docking domains are intertwined coiled-coil dimers (65, 66), thereby also contributing to module dimerization in addition to intermodular chain translocation. The C-terminal docking domain is composed of a dimeric coiled coil and a flexible linker connecting to helices that dock onto the N-terminal docking domains in an antiparallel orientation, creating a 4-helix bundle.

Proteins with docking domains are typically annotated with parentheses indicating their relative placement to a module. For example, when referring to components of DEBS, ACP2(2), ACP2(0), and (5)M3 correspond to ACP2 with a C-terminal docking domain from Module 2, ACP2 without a docking domain, and Module 3 with the N-terminal docking domain from Module 5, respectively.

### The Ketosynthase–Acyltransferase Core

The core of a typical module of a *cis*-AT assembly-line PKS is composed of a ~190-kDa homodimeric unit that includes the KS and AT domains flanked by the helical N-terminal docking domain; a second,well-structured,~100-residue linker subdomain between the KS and AT domains that represents a unique protein fold (the KS–AT linker); and a ~30-residue post-AT linker that wraps around both the AT and KS–AT linker domains to form extensive surface interactions with the KS domain. Although the primary amino acid sequences of these flanking regions are considerably more divergent than those of the KS or AT domains, their tertiary structures are conserved.

By now, the structures of KS–AT cores, referring to the full didomain spanning from the KS domain to the post-AT linker, of multiple PKS modules have been solved at 2.09–2.80-Å resolution (27, 57, 67–69). A representative structure is shown in Figure 4. Multiple structures of KS cores from *trans*-AT PKSs have also been solved (53, 70). These KS domains, which lack fused AT domains, have structurally conserved KS–AT linker subdomains with additional helical elements absent from *cis*-AT PKSs (Figure 2b). Crystallographic inspection of these helical elements from 10 different *trans*-AT KSs revealed conserved laterally interacting ketosynthase sequence (LINKS) motifs believed to be responsible for laterally grouping multiple PKS assembly lines into large megacomplexes inside the cell (71). Support for this hypothesis was recently obtained through cryo-EM analysis of a *trans*-AT PKS bimodule consisting of long filaments of sideways, interacting KS domains (72). Additionally, the discrete AT that interacts with *trans*-AT KS cores, such as the disorazole synthase AT, shows strong similarities with ATs found from *cis*-AT modules (Figure 2c) (73).

Although the KS–AT cores of DEBS Modules 1 (35), 2 (58), 3 (67), and 5 (27) and their homolog from Module 14 of the lasalocid synthase (Lsd14) (56) show strong architectural similarity to each other, their counterparts from Module 8 of the curacin synthase (68) and the loading module of the pikromycin synthase (69) show variable rotation of the AT domain, suggesting the KS–AT linker subdomain acts as a hinge. The mechanistic relevance of the degree of inward rotation is discussed below.

### The Reductive Loop

The KS–AT core and the C-terminal ACP domain of a typical PKS module are typically bridged by a structurally and functionally variable region that is referred to as the reductive loop on account of its ability to catalyze reductive modifications on the β-ketoacyl product of the elongation reaction. This region of a PKS module can be as short as 20 residues (e.g., Module 2 of the rifamycin synthase, which translocates an unmodified elongation product to Module 3) or as large as the KS–AT core itself (e.g., DEBS Module 4). Examples of catalytic domains housed in this region include the KR, DH, and ER domains, for which prototypical structures have been solved. The structure of the KR domain of DEBS1 (Figure 4) reveals two subdomains, one structural and one catalytic (54), whereas the structure of the DH domain of DEBS Module 4 (Figure 4) reveals a double-hotdog fold (74). A crystal structure of the KR–ER didomain from Module 2 of the spinosyn PKS has also been solved (75). Recently, the full reducing region (DH–ER–KR) of Module 5 of the juvenimicin PKS has been structurally characterized at atomic resolution (Figure 4) (55). Among other significant features, this prototypical structure reveals an architecture that is markedly different from that of the homologous DH–ER–KR segment of the mammalian FAS and a dimer interface formed exclusively between the two DH domains (Figure 2a).

### The Acyl Carrier Protein

The structure of the stand-alone ACP domain of DEBS Module 2 has been solved via solution NMR (Figure 4) (76). This ACP is composed of three α-helices and two unstructured loops, with several hydrophobic residues stabilizing the interactions between the helices. Homology modeling of all other ACP domains of DEBS suggests marked variability in electrostatic surfaces, which potentially play a role in the selectivity of KS–AT–ACP interactions (77). The relevance of these homology models has been reinforced by cryo-EM structures of larger protein systems harboring ACP domains from Modules 1 (35) and 3 (D.P. Cogan, A.M. Soohoo, M. Chen, Y. Liu, K.L. Brodsky, C. Khosla, manuscript in preparation) of DEBS.

### Thioesterase

The DEBS thioesterase (TE) domain was the first structurally elucidated component of an assembly-line PKS (Figure 4) (78). Its crystal structure revealed a dimeric architecture with subunits held together via predominantly hydrophobic interactions. A substrate channel passing through the entire protein was also observed, with several residues in the channel that plausibly formed hydrogen bonds with the substrate. All TEs show a similar fold, although the size of the substrate channel is variable (79).

### Intact Polyketide Synthase Modules

Although the aforementioned divide-and-conquer approach to structural characterization of assembly-line PKSs yielded reproducible, high-resolution insights into prototypical domains and didomains of individual PKS modules, until recently, the only structures of intact modules and bimodules were deduced by combining these atomic structures of module fragments with moderate-resolution data on the larger systems obtained via SEC-SAXS (80) or cryo-EM (81, 82). These early studies provided confirmation of the architectural robustness of PKSs in that modules can be taken apart or pieced together with equivalent structural insights. The study of Module 5 of the pikromycin PKS foreshadowed the significance of conformational dynamics of PKS modules as they proceed through their catalytic cycles (81, 82).

Using newer methods, we and others have recently reported single-particle cryo-EM structures of several intact modules at near-atomic resolution, including DEBS Module 1 (35) and Module 3 (D.P. Cogan, A.M. Soohoo, M. Chen, Y. Liu, K.L. Brodsky, C. Khosla, manuscript in preparation) as well as the more distantly related Module 14 of the lasalocid PKS (56). The same study also reported a crystal structure (2.35 Å) of this module obtained in the absence of 1B2 (Figure 2a). What is remarkable about most cryo-EM-derived structures is the asymmetry of the module architectures, despite previous models invoking module symmetry—based, in part, on C2-symmetric crystal structures of the architecturally related mammalian FAS (Figure 2a) (83, 84). As discussed below, the profound mechanistic relevance of asymmetric modular conformations is emphasized by their dominance in samples where transient catalytic states are trapped using different experimental strategies.

## MECHANISTIC PRINCIPLES OF ASSEMBLY-LINE POLYKETIDE BIOSYNTHESIS

While many biochemical and biophysical features of assembly-line PKSs have been investigated, in this section we limit our discussion to five properties of these remarkable multi-enzyme systems. We first focus on our current understanding of how extender unit specificity and stereospecificity are controlled; to a first approximation, these features are dictated by individual active sites. Thereafter, we turn our attention to examples where the property in question involves collaborative control by multiple domains of the assembly line.

### Extender Unit Transacylation

As discussed above, the AT domain of each PKS module installs onto its ACP partner an α-carboxyacyl extender unit, which in turn serves as the nucleophilic substrate for the KS-catalyzed C–C bond–forming elongation reaction (Figure 3). Due to the modest specificity of the KS domain for its extender unit substrate (85), the active site of the AT domain is primarily responsible for the extender unit specificity of a module. This property of each module of an assembly-line PKS has been subjected to strong evolutionary pressure during the structural diversification of polyketide natural products. Indeed, nature may have coopted some unusual evolutionary mechanisms to rapidly diversify extender unit selection. For example, many assembly-line PKSs harbor GRINS (genetic repeats of intense nucleotide skews) that appear to have enabled late-stage AT diversification in closely related assembly-line PKSs (86).

**Figure 5:**
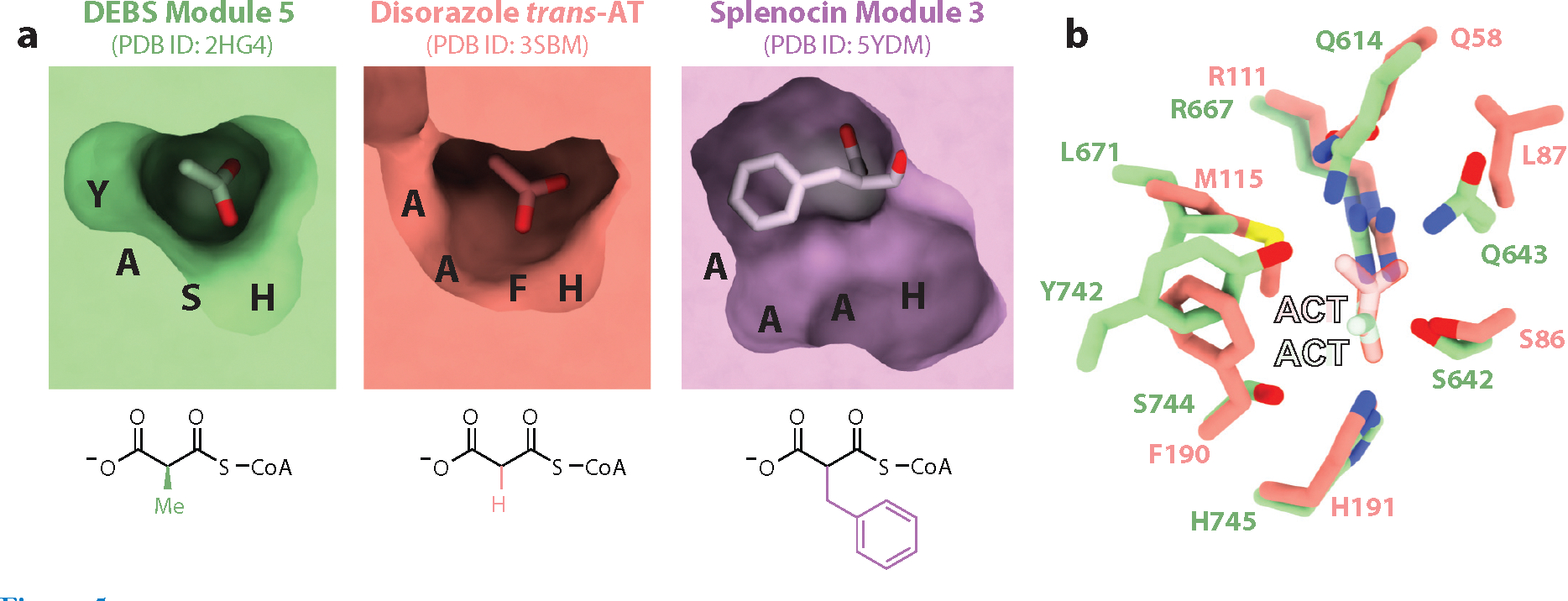
Active sites of representative ATs. (*a*) Cross section of the active sites of different ATs, including the AT from DEBS Module 5 (27), the disorazole synthase *trans*-AT (73), and the AT from Module 3 of the splenocin synthase (90), with associated extender units indicated below. Residues comprising the extender unit specificity motifs (i.e., YASH, AAFH, and AAAH) are shown on the surface to highlight their roles in pocket size and substrate recognition. ACT ligands mimicking the carboxylate moieties of native extender units were crystallographically observed in the enzyme active sites of the DEBS AT (PDB ID: 2HG4) and the disorazole AT (PDB ID: 3SBM). The crystal structure of splenocin AT was determined in covalent complex with a benzylmalonyl extender unit (PDB ID: 5YDM), corresponding to the native acyl-enzyme intermediate that precedes transfer of the extender unit onto the ACP. (*b*) Superposition of the DEBS AT and disorazole AT active sites highlights their similarities and differences in terms of size and chemical properties. Abbreviations: ACT, acetate; AT, acyltransferase; DEBS, 6-deoxyerythronolide B synthase; PDB ID, Protein Data Bank identifier.

All ATs from *cis*- and *trans*-AT PKSs have a serine protease–like active site. For example, in the disorazole AT shown in Figure 5, S86 and H191 contribute the nucleophile and general base, respectively. The extender unit is transiently acylated onto the nucleophilic hydroxyl during transfer from a selected CoA metabolite onto an ACP. Given the presence of a Ppant substituent on both CoA and ACP substrates of an AT, a ping-pong mechanism can be anticipated for this family of enzymes. While DEBS AT domains do in fact operate via a ping-pong bi–bi mechanism (87), stand-alone ATs such as the disorazole AT require both substrates to bind before the CoASH leaving group dissociates, thereby allowing molecular recognition features for both substrates to be expressed in the overall specificity (*k*_cat_/*K*_M_) of these enzymes (88).

The extender unit and ACP specificity of several representative *cis*-ATs and stand-alone ATs have been quantified. Most ATs also show some, albeit not high (~10-fold) (89), preference for their cognate ACP substrates. In contrast, they show higher specificity (100- to 10,000-fold) for their cognate carboxyacyl-CoA substrates (87), highlighting a gatekeeper role for this family of enzymes during polyketide biosynthesis. What is noteworthy is their ability to differentiate between relatively similar substrates; for example, the methylmalonyl-specific AT domain of DEBS Module 3 can discriminate against both malonyl-CoA and ethylmalonyl-CoA (88).In most cases, while this discriminant capacity is inbuilt in the *k*_cat_ and *k*_cat_/*K*_M_, certain ATs, such as the ethylmalonyl-specific KirCII, appear to have evolved toward optimization of *K*_M_ only.

The structural basis for the extender unit specificity of individual ATs remains an ongoing area of investigation, despite available structural information regarding the AT active site of DEBS (27), disorazole (73), and splenocin synthases (90). While some control is undoubtedly exercised by a substrate-binding pocket near the active site (Figure 5), more distal residues are also known to influence specificity, suggestive of an evolutionarily complex and potentially divergent strategy for selecting the correct building block (91–93). Sequence analysis has identified relatively well-conserved sequences associated with extender unit specificity [i.e., the YASH sequence with methylmalonyl extender units and the HAFH sequence with malonyl extender units (93)], although conformational dynamics are also likely to play a role (94). The same conclusion can be drawn with regard to ACP specificity. For example, the structure of Module 14 from the lasalocid A synthase revealed its ACP docked onto the AT predominantly via Helix II (56). In contrast, the X-ray structure of a cross-linked AT–ACP pair from the salinomycin synthase revealed significant rotational differences in the ACP relative to its lasalocid counterpart (95). Similar differences in docking pose have also been discerned in the interactions of *trans*-AT enzymes with their ACP substrates, as illustrated by a comparison of a representative AT–ACP pair from the disorazole synthase (96) and the vicenistatin synthase (97).

### Stereochemistry

Stereochemical control and diversity are arguably among the most remarkable features of the polyketide products of assembly-line PKSs. For example,6-dEB has 10 stereocenters and ~10^3^ diastereomers, yet the product of DEBS exhibits unique chirality. As early as the 1960s, Celmer (98) observed that macrolide antibacterials share stereochemical features, suggesting genetic control of this structural aspect. The discovery of the assembly-line PKS paradigm prompted reevaluation of the enzymatic basis for stereocontrol, a topic that has been reviewed in depth (99, 100).

**Figure 6:**
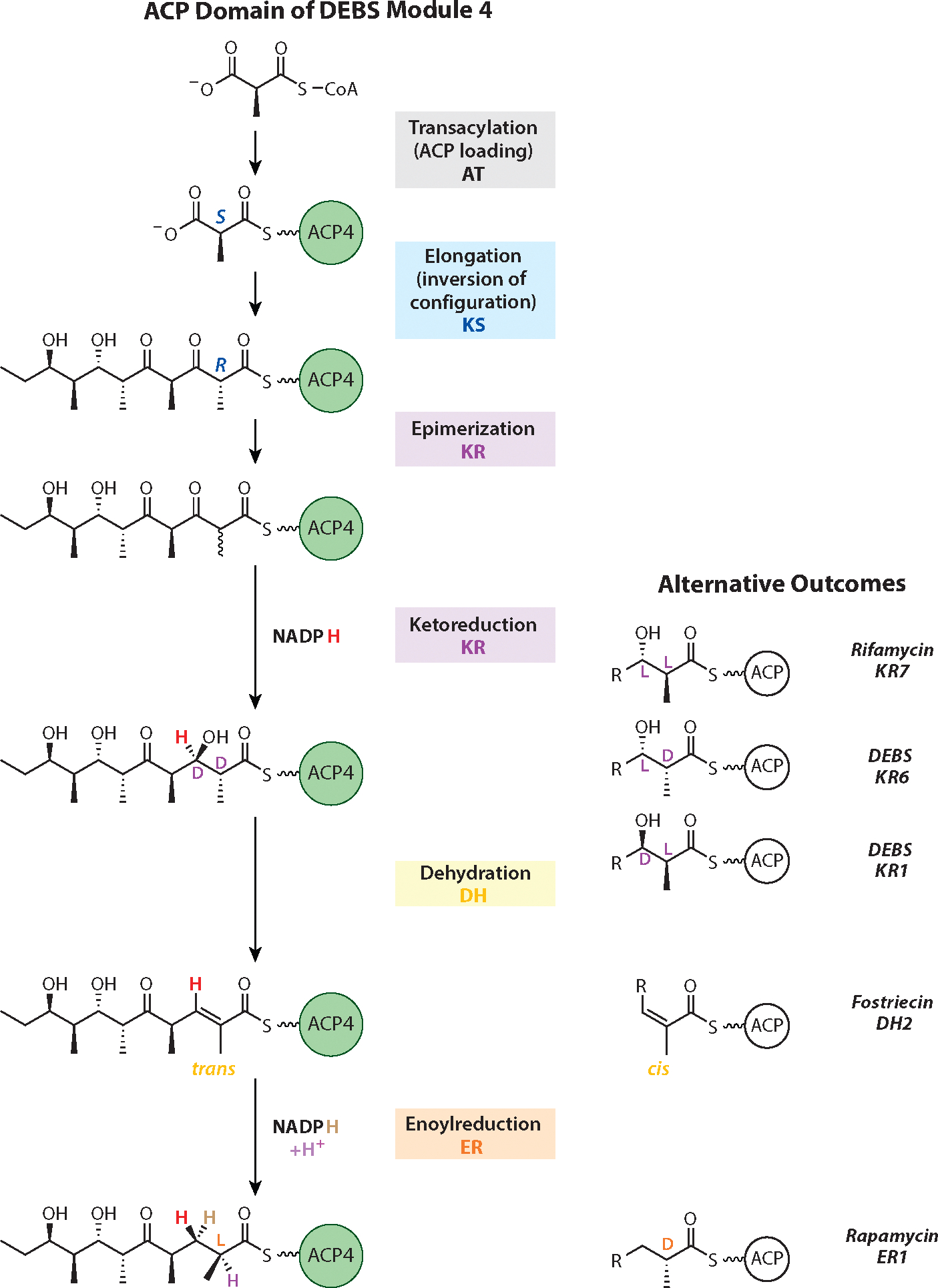
Alternative stereochemical outcomes of reactions catalyzed by reductive loop enzymes. The left column shows the stereochemical fates of the sequence of reactions catalyzed by DEBS Module 4. Stereochemical diversity observed at equivalent steps in other representative modules is shown in the right column. Abbreviations: ACP, acyl carrier protein; AT, acyltransferase; DEBS, 6-deoxyerythronolide B synthase; DH, dehydratase; ER, enoylreductase; KR, ketoreductase; KS, ketosynthase.

Because all DEBS modules use (2*S*)-methylmalonyl-CoA as an extender unit (101), it follows that individual stereocenters of polyketide products such as 6-dEB are dictated either by the KS domain during the chain elongation step or by the reductive loop enzymes during postelongation modifications (Figure 6). Following the discovery that the Claisen-like condensation reaction invariably results in stereoinversion (102), the need for an α-methyl epimerase in Modules 1 and 3 of DEBS became apparent. Once it became possible to dissociate PKS modules into their functional catalytic components (Figure 4), the source of this epimerase activity could be localized to the KR domains of the corresponding modules, which were shown to catalyze epimerization of the α-methyl group prior to reduction (103). Thus, KR domains control the diastereochemical outcome of the β-ketoreductive step by programming the stereochemistry of both the α- and β-carbon atoms (104). Because a DH can dehydrate its substrate (105), this family of enzymes is responsible for generating a double bond with a *trans* or *cis* geometry. Thus, the DH domains of rifamycin Module 10 (106) and fostriecin Module 2 (107) generate distinct α,β-unsaturated esters. In addition, an ER can reduce the alkene to an alkane with the resulting methyl group having either an *S* or *R* configuration. A conserved tyrosine has been implicated in this stereochemical control, although other residues are likely involved (108). The known degrees of freedom in stereochemical control exercised by assembly-line PKSs are summarized in Figure 6.

### Module Specificity

Although the pairwise sequence identities of the KS–AT–ACP cores of the six DEBS modules range between 40–50% for ACPs and 50–65% for KS–AT fragments, their order of usage is uniquely defined. Textbook models for metabolic specificity might suggest that the active site of each KS has evolved elaborate molecular recognition features to allow only translocation of its cognate acyl chain substrate. However, in the few instances where KS specificity has been systematically interrogated, this has not been found to be the case. For example, the natural diketide substrate of DEBS Module 2 (Figure 3) shows 40% and 140% selectivity (defined as relative *k*_cat_/*K*_M_ values of the same substrate for alternative enzymes) for Modules 3 and 6, respectively, when compared to its cognate acceptor module (45). This is not to suggest that KS domains are nonspecific catalysts. For example, the KS domains of all three modules exhibit ~100-fold higher specificity for NDK-SNAC (Figure 3) compared to its enantiomer. Moreover, the relative specificity of individual KS domains for a panel of alternative acyl chains is unaffected by the identity of the leaving group (i.e., SNAC or ACP) (44), suggesting that substrate preference is an inherent property of KSs. Nonetheless, the time-honored principle that the overall specificity of a metabolic pathway is a product of the specificity of individual active sites fails to explain why the product of DEBS Module 1 is not channeled to either Module 3 or Module 6 at a measurable frequency.

**Figure 7:**
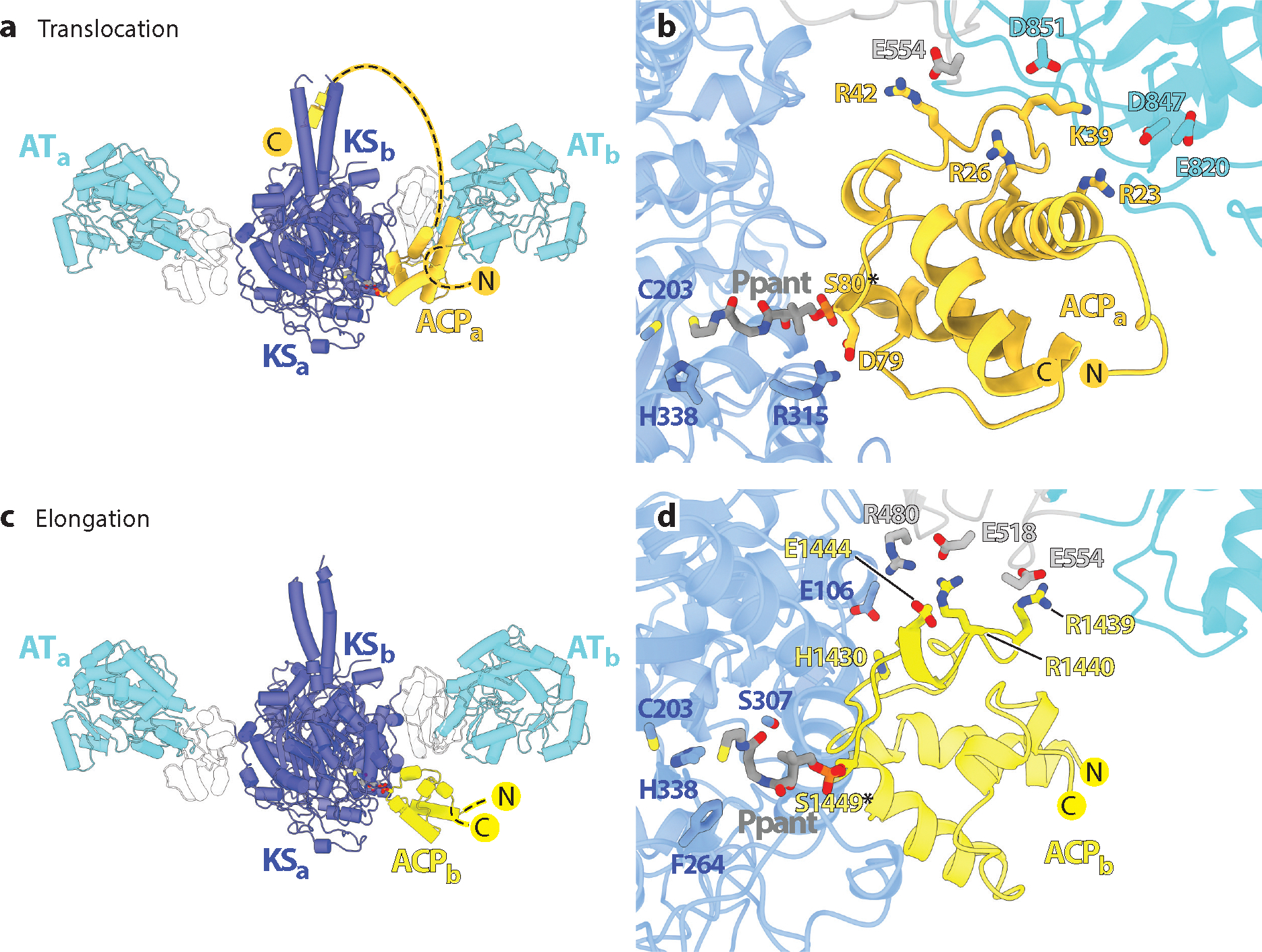
KS–AT and ACP interactions. (*a*) Structure of an upstream module ACP (DEBS ACP2) interacting with its cognate KS–AT (from Module 3) in a translocation-relevant state (D.P. Cogan, A.M. Soohoo, M. Chen, Y. Liu, K.L. Brodsky, C. Khosla, manuscript in preparation). (*b*) Residue-level interactions between the ACP and KS–AT during chain translocation. (*c*) Structure of the intramodular ACP (DEBS ACP3) interacting with its cognate KS–AT (from Module 3) in an elongation-relevant state (D.P. Cogan, A.M. Soohoo, M. Chen, Y. Liu, K.L. Brodsky, C. Khosla, manuscript in preparation). (*d*) Residue-level interactions between the ACP and KS–AT during chain elongation. Abbreviations: ACP, acyl carrier protein; AT, acyltransferase; DEBS, 6-deoxyerythronolide B synthase; KS, ketosynthase; Ppant, 4′-phosphopantetheine.

A substantial body of evidence supports a more significant role for protein–protein interactions in controlling the module order of a PKS assembly line (109). Two types of repetitive interdomain interactions are especially important (Figure 7). First, as discussed in the section titled Docking Domains, in situations where successive modules require noncovalent interactions, docking domains stabilize these interactions. Docking domain interactions are separably distinct from a second type of protein–protein interaction involving the KS–AT cores of individual modules, which exhibit dynamic specificity for their cognate ACP partners (Figure 7) (44, 64, 110). As each module proceeds through its catalytic cycle (Supplemental Video 1), the KS–AT core first recognizes its upstream ACP partner during the translocation step (Figure 7a) and then docks with its intramodular ACP partner during the elongation step (Figure 7c). Notwithstanding the high sequence similarity between individual ACP domains, their docking orientations are different in these two steps (110). Furthermore, in each step, the protein–protein interface appears to rely on multiple steric as well as electrostatic interactions to preclude noncognate KS–AT–ACP interactions (Figure 7b,d) (77). The magnitude of ACP specificity is not extraordinarily high (~10-fold preference for cognate KS–AT–ACP pairs) in both translocation and elongation reactions; however, in conjunction with covalent or docking domain–mediated mechanisms it appears to provide an adequately strong filter to ensure that modules are used in a uniquely defined order.

As is perhaps most vividly illustrated by the example of antibody evolution, nature has presumably harnessed elaborate missense and indel mutagenesis mechanisms to fine-tune intermodular interfaces of evolutionarily promising assembly lines that may have arisen via module recombination. Now that the principles of KS–ACP recognition are being defined at a structural level, one can hope that it will be possible to define the analogs of immunoglobulin complementarity-determining regions in PKS modules.

### Vectorial Biosynthesis

If successive modules of the DEBS assembly line were aligned in an end-to-end manner, this assembly line might stretch across ~1,000 Å. How is vectorial biosynthesis achieved along a uniquely defined path that includes thirteen alternating ACP and KS thiols and requires the growing polyketide chain to cross the dimer interface six times?

A powerful conceptual framework to understand the mechanistic basis for directional biosynthesis on an assembly-line PKS can be adapted from William P. Jencks’s (111) insightful analysis of the coupled vectorial processes underlying actomyosin-based muscle contraction and ATP-dependent ion pumps. Jencks articulated two key features of unidirectional catalytic machines. First, they exhibit a geometric change in substrate specificity as they traverse their catalytic cycles; the geometric aspect of this specificity change constrains the system to move forward while preventing it from reverting to its original state. Second, the exergonicity of ATP hydrolysis promotes this flip in specificity; thus, like machines in the macroscopic world, biomolecular machines utilize chemical energy to perform work.

Assembly-line polyketide biosynthesis displays both these hallmark features of coupled vectorial processes. As discussed above, the KS–AT core of each module toggles between its translocation state, in which it preferentially recognizes its upstream ACP partner, and its elongation state, in which it specifically binds to the ACP domain from its own module. Furthermore, whereas the vectorial nature of polyketide biosynthesis on an assembly-line PKS is enabled by two distinct translocation events in each module (Figure 3), both these reactions are thermoneutral (thiol-to-thioester exchange reactions) and must therefore be coupled to a bridging exergonic reaction, namely the Claisen-like condensation, which is presumably the power stroke that flips the ACP specificity of the KS–AT core.

**Figure 8:**
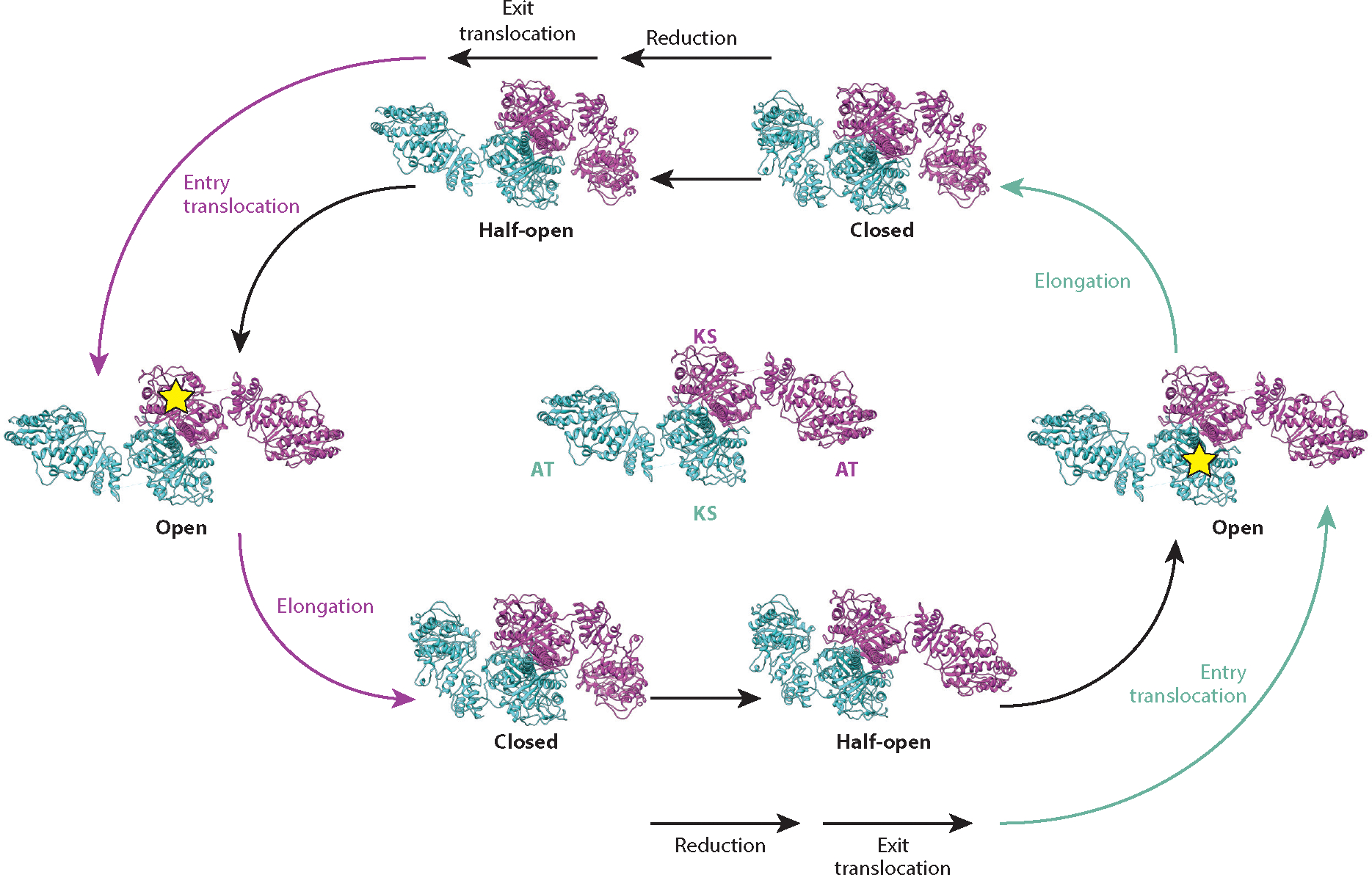
Model for conformational changes in the KS–AT core over the catalytic cycle. Individual domains of a representative homodimeric conformer are labeled in the center; in all states shown, one monomer is shown in magenta, and the other is shown in cyan. Each conformer has been observed in one or more near-atomic cryo–electron microscopy structures (35) (D.P. Cogan, A.M. Soohoo, M. Chen, Y. Liu, K.L. Brodsky, C. Khosla, manuscript in preparation). A yellow star on a KS domain implies that its active site is occupied with its substrate. When a KS–AT subunit assumes a turnstile-open conformation (shown at the far left and right of the catalytic cycle), the wide cleft between the KS and AT domains is large enough to accommodate either the upstream ACP associated with translocation or the internal ACP associated with elongation. Two equivalent turnstile-closed states and two half-open states are also shown. In the closed state, the KS–AT cleft is too narrow to allow an ACP to dock onto the KS in a catalytically productive manner. For additional details regarding this view of the catalytic cycle including relevant KS–ACP interactions, see Supplemental Video 1. Abbreviations: ACP, acyl carrier protein; AT, acyltransferase; KS, ketosynthase.

To harness some of the free energy of the elongation reaction to alter geometric specificity, PKS modules have evolved an elegant mechanism to toggle each of two ACP-binding clefts in the KS–AT core between a conformation favoring the upstream ACP (Figure 7a) and a subtly different one favoring the ACP from the same module (Figure 7c). Dubbed the turnstile mechanism, this subtle conformational change occurs via a more profoundly altered intermediate state (called the turnstile-closed state) during which both KS active sites are inaccessible to either ACP partner (112). Cryo-EM analysis has revealed that, in this turnstile-closed state, the outstretched KS–AT conformer (called the turnstile-open state) flexes its AT domains, leading to a dramatic shrinkage of both ACP-binding clefts (35). A semiopen conformation has also been observed via cryo-EM analysis, suggesting that the two clefts open asynchronously. Together, these observations can be combined with earlier findings to visualize the catalytic cycle shown in Figure 3 through the lens of the homodimeric KS–AT core (Figure 8).

In addition to allowing the translocation state of a PKS module to be energetically coupled to its elongation state, a second important corollary of the turnstile mechanism is that the newly elongated polyketide chain has ample time for reductive loop modifications without risking back-translocation to a KS. (As discussed in the section titled Catalytic Cycle of a Polyketide Synthase Module, the inability of a polyketide chain to undergo back-translocation is the signature difference between assembly-line PKSs and all other members of the FAS and PKS superfamilies.) When the turnstile reopens, it first favors docking of the upstream ACP so that the KS active site can be replenished with a fresh equivalent of its polyketide substrate.

To verify that turnstile closing is energetically coupled to chain elongation and not merely an allosteric consequence of the ACP harboring a newly elongated polyketide chain, a PPTase was used to load the diketide product of DEBS Module 1 directly onto its ACP domain (112). In contrast to Module 1, which generated the chemically identical species via decarboxylative condensation between its KS-bound propionyl species and the ACP-bound methylmalonyl extender unit followed by β-ketoreduction, the KS domain of the PPTase-modified Module 1 remained accessible to another equivalent of a propionyl substrate. Thus, Jencks’s model for the utilization of binding energy in vectorial polyketide biosynthesis is manifested via an elongation-promoted geometric change in specificity of the KS–AT core.

### Cooperativity

As mentioned above, the homodimeric nature of assembly-line PKSs begs the question of whether pairs of active sites exhibit positive, negative, or no cooperativity. Cross-linking and cryo-EM analysis of intact DEBS Modules 1 and 3 in the presence of their cognate upstream ACP domains has revealed that simultaneous occupancy of both KS active sites of the homodimer by two equivalents of the same ACP partner is disfavored, both for the upstream (translocation) ACP and for the internal (elongation) ACP (D.P. Cogan, A.M. Soohoo, M. Chen, Y. Liu, K.L. Brodsky, C. Khosla, manuscript in preparation). In contrast, simultaneous occupancy of the two ACP-binding clefts by different ACPs, while not especially favorable, is also not prohibited. Thus, negative cooperativity is an intrinsic feature of a module during both the translocation and elongation steps of the catalytic cycle, a finding that is consistent with the model shown in Figure 8.

## AN INTEGRATED MODEL FOR ASSEMBLY-LINE POLYKETIDE BIOSYNTHESIS

Tying together all the insights gained through structural and mechanistic analyses of selected modules of DEBS, a model begins to emerge for visualizing key aspects of assembly-line polyketide biosynthesis (Supplemental Video 1). Building on the protein-level catalytic cycle shown in Figure 8, our Supplemental Video could start in the state shown at the far right of this scheme, where the module is in its turnstile-open state. One of the two KS active sites is acylated with the growing polyketide chain while the ACP of the other monomer is acylated with a methylmalonyl extender unit. The system is set up for an intersubunit elongation event whose free energy is used to induce the high-energy turnstile-closed conformation of the homodimer with concomitant ejection of the ACP-bound, freshly elongated polyketide product. As this elongated chain undergoes β-ketoacyl group modifications amid the reductive loop enzymes, the turnstile-closed state of the KS–AT core relaxes via a semiopen intermediate state that is translocation competent. At this point, the system is in a functionally equivalent state to where our Supplemental Video started, except that the opposite KS is occupied with a growing polyketide chain.

By coupling exergonic elongation to thermoneutral entry translocation, the module shown in our Supplemental Video ensures faithful toggling between specificity for the upstream and internal ACP domains, thereby satisfying Jencks’s postulates for vectorial protein machines. Such coupling not only can be expected to enforce directionality of polyketide biosynthesis along the assembly line but also would explain why disruptions to KS–AT–ACP interfaces can have profoundly negative kinetic consequences—a well-known observation among PKS engineers.

## FUTURE DIRECTIONS

In-depth analysis of DEBS and a handful of other assembly-line PKSs has provided significant insights into the architectures and mechanisms of this multifunctional enzyme family. While some problems, such as extender unit control or stereospecificity, have been reduced to classical challenges in enzymology whose solutions lie in a deeper understanding of individual active sites, other problems, such as vectorial biosynthesis, are systems-level properties of assembly lines.

Notwithstanding these advances, key aspects of assembly-line PKS enzymology elude a satisfactory explanation. For example, one cannot examine the sequence or structure of an assembly-line PKS to infer the order of module usage or explain how the growing polyketide chain is extracted from each KS active site and delivered to the next one via the translocation step. The frequently occurring interactions between assembly-line PKSs and the related nonribosomal peptide synthetases (NRPSs) present an especially intriguing class of mechanistic problems. As just one example, how is cooperativity manifested, given their different oligomerization states in catalysis (NRPS modules usually function as monomers, while PKS modules are dimers) (113)? From a practical perspective, an enhanced understanding of AT specificity, as well as the stereospecificity of reductive loop enzymes, could be harnessed for innumerable biosynthetic engineering applications. The final frontier for this family of enzymes is no different from the “far reaches of enzymology” described by Zalatan & Herschlag (114). Specifically, it is now well understood that advances in probing the link between enzyme structure and function can pave the way for a deeper understanding of the evolution of not only these enzyme families themselves but also the more complex biological systems within which they operate. In that spirit, much like the advances that have occurred in antibody science and technology over the past four decades, our understanding of the structural basis for the unusually rapid functional diversification of assembly-line PKSs promises to herald a new era in antibiotic discovery and engineering. What is abundantly clear is the continued need for new tools and methodologies to advance our understanding of these remarkably versatile catalytic systems.

## Supplementary Material

Movie 1
